# Applying Statistical Design of Experiments To Understanding the Effect of Growth Medium Components on Cupriavidus necator H16 Growth

**DOI:** 10.1128/AEM.00705-20

**Published:** 2020-08-18

**Authors:** Christopher C. Azubuike, Martin G. Edwards, Angharad M. R. Gatehouse, Thomas P. Howard

**Affiliations:** a School of Natural and Environmental Sciences, Faculty of Science, Agriculture and Engineering, Newcastle University, Newcastle-upon-Tyne, United Kingdom; b Department of Microbiology, Faculty of Science, University of Port Harcourt, Port Harcourt, Rivers State, Nigeria; Kyoto University

**Keywords:** *Cupriavidus necator* H16, design of experiments (DOE), automation, chemically defined media

## Abstract

Cupriavidus necator H16 is gaining significant attention as a microbial chassis for range of biotechnological applications. While the bacterium is a major producer of bioplastics, its lithoautotrophic and versatile metabolic capabilities make the bacterium a promising microbial chassis for biofuels and chemicals using renewable resources. It remains necessary to develop appropriate experimental resources to permit controlled bioengineering and system optimization of this microbe. In this study, we employed statistical design of experiments to gain understanding of the impact of components of defined media on C. necator growth and built a model that can predict the bacterium’s cell density based on medium components. This highlighted medium components, and interaction between components, having the most effect on growth: fructose, amino acids, trace elements, CaCl_2_, and Na_2_HPO_4_ contributed significantly to growth (*t* values of <−1.65 or >1.65); copper and histidine were found to interact and must be balanced for robust growth. Our model was experimentally validated and found to correlate well (*r*^2^ = 0.85). Model validation at large culture scales showed correlations between our model-predicted growth ranks and experimentally determined ranks at 100 ml in shake flasks (ρ = 0.87) and 1 liter in a bioreactor (ρ = 0.90). Our approach provides valuable and quantifiable insights on the impact of medium components on cell growth and can be applied to model other C. necator responses that are crucial for its deployment as a microbial chassis. This approach can be extended to other nonmodel microbes of medical and industrial biotechnological importance.

**IMPORTANCE** Chemically defined media (CDM) for cultivation of C. necator vary in components and compositions. This lack of consensus makes it difficult to optimize new processes for the bacterium. This study employed statistical design of experiments (DOE) to understand how basic components of defined media affect C. necator growth. Our growth model predicts that C. necator can be cultivated to high cell density with components held at low concentrations, arguing that CDM for large-scale cultivation of the bacterium for industrial purposes will be economically competitive. Although existing CDM for the bacterium are without amino acids, addition of a few amino acids to growth medium shortened lag phase of growth. The interactions highlighted by our growth model show how factors can interact with each other during a process to positively or negatively affect process output. This approach is efficient, relying on few well-structured experimental runs to gain maximum information on a biological process, growth.

## Abstract (precis)

Chemically defined media (CDM) for cultivation of C. necator vary in components and compositions. This lack of consensus makes it difficult to optimize new processes for the bacterium. This study employed statistical design of experiments (DOE) to understand how basic components of defined media affect C. necator growth. Our growth model predicts that C. necator can be cultivated to high cell density with components held at low concentrations, arguing that CDM for large-scale cultivation of the bacterium for industrial purposes will be economically competitive. Although existing CDM for the bacterium are without amino acids, addition of a few amino acids to growth medium shortened lag phase of growth. The interactions highlighted by our growth model show how factors can interact with each other during a process to positively or negatively affect process output. This approach is efficient, relying on few well-structured experimental runs to gain maximum information on a biological process, growth.

## INTRODUCTION

Cupriavidus necator H16 is of biotechnological importance largely due to its ability to accumulate >80% of its dry cell weight as polyhydroxyalkanoate (PHA)—a biodegradable polymer and an alternative to petroleum-based polymers (1, 2). PHA (specifically 3-polyhydroxybutyrate [3-PHB]) accumulation by the bacterium is a carbon conservation mechanism. When nitrogen, oxygen, or phosphorus becomes limiting to cell growth, the bacterium diverts excess carbon to 3-PHB. This carbon store supports growth when conditions improve (3–6). C. necator is also chemolithoautotrophic, with the ability to use CO_2_ and formate or H_2_ as carbon and energy sources to support cellular metabolism (7). In the absence of oxygen, it can use alternative electron acceptors (NO_3_^−^ and NO_2_^−^) to carry out anaerobic respiration by denitrification (8). More widely, the genus is known to possess genes facilitating metabolism of environmental pollutants such as aromatics and heavy metals, making it a potential microbial remediator (9–12). C. necator is central to efforts to construct systems for the electromicrobial conversion of CO_2_ to higher alcohols (7). Metabolic engineering of C. necator has been demonstrated through the introduction of pathways for the biosynthesis of alcohols (6, 7, 13, 14), fatty acids (15–17), alkanes (18), and enzymes (19) under both heterotrophic and autotrophic growth conditions. The production of branched-chain alcohols by C. necator through electricity-powered cellular synthesis (i.e., electrosynthesis) demonstrates the value of this bacterium as a chassis capable of exploiting renewable feedstock for the biosynthesis of valuable products (7). As with any bacterium, the development of C. necator as an industrial chassis requires appropriate tools for studying and engineering the organism. One of these resources is the availability of a characterized, chemically defined growth medium. Chemically defined media (CDM) are important to enable experimental reproducibility, to reliably characterize the genetics of the organism, to determine genotype by environment interactions, and to facilitate fundamental research of bacterial physiology that underpins bioengineering efforts. While different chemically defined media have been deployed for the cultivation of C. necator (3, 13, 14, 16, 20–22), there is no consensus regarding the components that are required, the concentration of each component, or how each component interacts to affect growth of the bacterium (see Table S1 in the supplemental material). This is not surprising given the range of industrial applications with the bacterium.

Design of experiments (DOE) is an iterative, empirical approach that systematically explores the relationship between input variables (factors) and output variables (responses). The approach yields a structured set of data that can be used to build statistical models employed in understanding or optimizing system performance. These statistical models can be validated against prior knowledge or internal statistical methods or, ultimately, by their ability to predict responses from new combinations of factors. The increasing availability of laboratory automation and high-throughput technologies may therefore be resulting in a greater appreciation for its application in bioengineering. DOE has found use in the optimization of metabolic pathways (23, 24), cell-free protein synthesis reactions (25), and codon use algorithms (26). It has been applied to the study of genotype-by-genotype and genotype-by-environment interactions in Saccharomyces cerevisiae (27) and in repurposing enzyme activities (28).

In this study, we employed a statistical engineering approach to build a data-driven model that can accurately predict C. necator growth responses to a range of medium formulations. The model highlighted different formulations that allow reproducible and robust growth of C. necator with the minimal concentration of each component and allowed us to identify and understand interactions between components of the media. Additionally, the model allowed the learning from the small scale (1 ml) to be applied at larger volumes (100 ml and 1 liter). Understanding the impact of each component of a chemically defined medium on C. necator growth is a fundamental tool for controlled exploration of the biotechnological potential of this important bacterium.

## RESULTS

### Identifying main ingredients in chemically defined media that affect the growth of C. necator.

**FIG 1:**
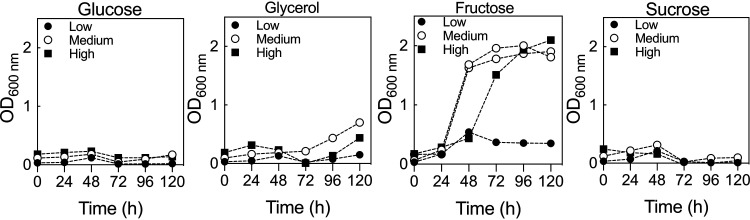
Scoping trials. Shown is growth of C. necator H16 in a 48-well format with different carbon sources. Each experiment was carried out at low, medium (*n *= 2), or high concentrations of each medium component (details can be found in Table 1).

The preliminary phase of the experiment was to determine whether optical density at 600 nm (OD_600_) is appropriate for determining C. necator growth. To establish this, the bacterium was cultivated in a rich medium (LB), and correlation between the CFU per milliliter and the OD_600_ was determined (Fig. S1). Growth obtained from optical density measurements correlated well with the number of viable cells. Next, an initial scoping trial was carried out using fructose, glucose, glycerol, or sucrose to identify a principal carbon source to be used for subsequent work and to determine the range of concentrations to be tested. Four scoping trials were performed, one each at low and high concentrations of medium components and two trials at the midpoint values between the two extremes (Table 1). At the ranges tested, glucose, glycerol, and sucrose supported little or no growth of C. necator, while fructose supported high growth except at low concentrations (Fig. 1). The OD_600_ for the two midpoint experiments demonstrated a peak at 72 h, followed by a plateau. From these scoping trials, it was established that fructose would be our principal carbon source, OD_600_ was an appropriate measure of cell growth, growth assays in 1-ml volumes in a 48-well plate format were appropriate for subsequent experiments, and recording OD_600_ at 72 h provided a good balance between measuring growth rate and peak culture density.

**TABLE 1:** Scoping experiment^*a*^

Medium	Concn of:									
	Carbon	NaH_2_PO_4_	Na_2_HPO_4_	K_2_SO_4_	MgSO_4_	CaCl_2_	NH_4_Cl	T.E.	A.A.	Vit.
Low	0.5	0.1	0.1	0.01	0.01	0.01	0.01	0.01	0.05	0.01
Medium	20.25	3.05	3.05	2.41	1.41	0.41	2.41	2.41	9.53	2.41
High	40	6	6	4.8	2.8	0.8	4.8	4.8	19	4.8

a A scoping trial was developed to assess the impact of 10 basic components of chemically defined media. All concentrations are in grams per liter, except for trace elements, amino acids, and vitamins, which are in milliliters per liter. Abbreviations: T.E., trace element mixture; A.A., amino acid mixture; Vit., vitamin mixture. Trace element working solution contained 15 g/liter of FeSO_4_·7H_2_O, 2.4 g/liter of MnSO_4_·H_2_O, 2.4 g/liter of ZnSO_4_·7H_2_O, and 0.48 g/liter of CuSO_4_·5H_2_O. A 100× stock amino acid mix contained 12.9 g/liter of arginine and 10 g/liter each of histidine, leucine, and methionine. A 1,000× vitamin stock contained 0.1 g/liter of pyridoxine, 0.02 g/liter of folic acid, and 0.05 g/liter each of thiamine, riboflavin, niacin, pantothenic acid, and nicotinamide. The carbon source was fructose, glucose, glycerol, or sucrose. The medium trial was performed in duplicate.

**FIG 2:**
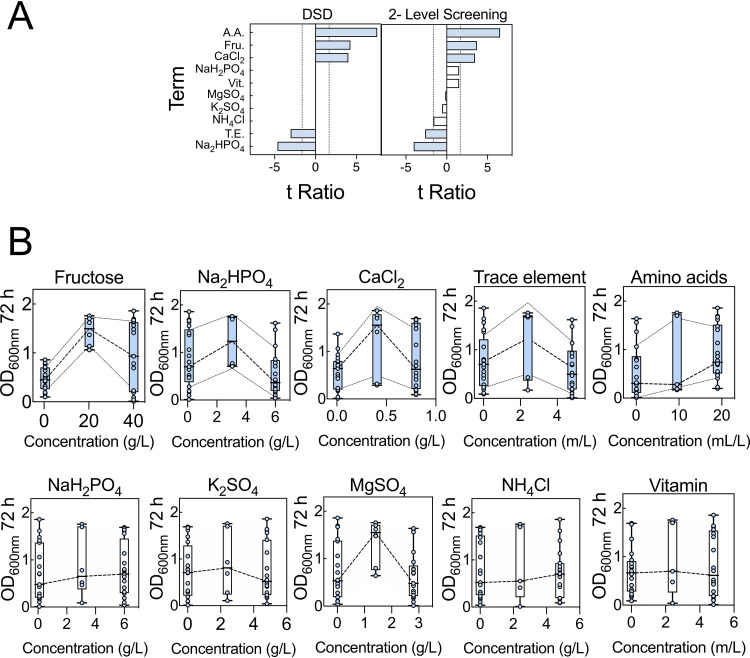
Definitive screening design array analysis. (A) Definitive screening and 2-level screening of data within DSD1 were performed. The comparative lengths of the *t* ratios for each factor and factor interaction are shown. At high concentrations, bars extending to the right have a positive impact on growth, while those extending to the left have a negative impact on growth. Terms deemed significant for model projection are shaded (blue). Abbreviations: Fru., fructose; T.E., trace element mixture; A.A., amino acid mixture; Vit., vitamin mixture. The broken vertical lines indicate threshold (*t* < −1.65 or *t* > 1.65) level at 90% confidence level. (B) Summary of main effect plot for each factor. The middle broken lines are connecting mean growth for each factor settings. Additionally, for significant factors, the broken lines above and below that of the mean growth are standard deviation bands. Error bars indicate SEMs. Analysis is based on two biological replicated arrays (Table S2).

We next identified key factors that might influence OD_600_ at 72 h. An initial definitive screening design (DSD1) array was built based on 10 medium components (Table S2). The compositions of the media in DSD1 are within the ranges indicated in Table 1. DSDs are highly efficient experimental designs in which all main effects can be estimated independently of other main effects and all possible two-way interactions. The requisite variant medium compositions were assembled in a 48-well plate and growth was monitored. Both analyses indicated that high concentrations of fructose, CaCl_2_, and amino acids contributed positively to growth, while high concentrations of disodium phosphate (Na_2_HPO_4_) and trace elements contributed negatively to growth. Factors such as NaH_2_PO_4_, K_2_SO_4_, MgSO_4_, NH_4_Cl, and vitamins were not found to have statistically significant effects under the conditions tested (Fig. 2A). These analyses also highlighted several two-way interactions that may influence growth responses (Fig. S2).

Besides the main effect of each component (factor), and interactions between some components of media, the mean growth distribution between factor settings (low, middle, and high) showed interesting responses (Fig. 2B). For example, the mean growth distribution for fructose showed a nonlinear (quadratic) effect, arguing that the optimum fructose concentration that will result in robust and less variable growth lies above 0.5 g/liter and below 40 g/liter. Na_2_HPO_4_ showed a nonlinear effect, with growth at the low setting reaching higher OD_600_ than at the high setting. Similarly, CaCl_2_ showed a nonlinear effect; the optimum concentration range for robust growth appears to lie above the low setting (0.01 g/liter) and below the high setting (0.8 g/liter). Trace elements also displayed a nonlinear effect. Amino acid data suggested that the optimum concentration was yet to be tested. Nevertheless, we avoided increasing the amino acid concentration given that this contributes to extra carbon and nitrogen to the media. The nonsignificant factors displayed linear effect, except MgSO_4_. Therefore, this initial DSD needs augmentation to establish an optimum concentration range for each component, especially components having the most significant effect on growth.

### Augmentation of the data set.

**FIG 3:**
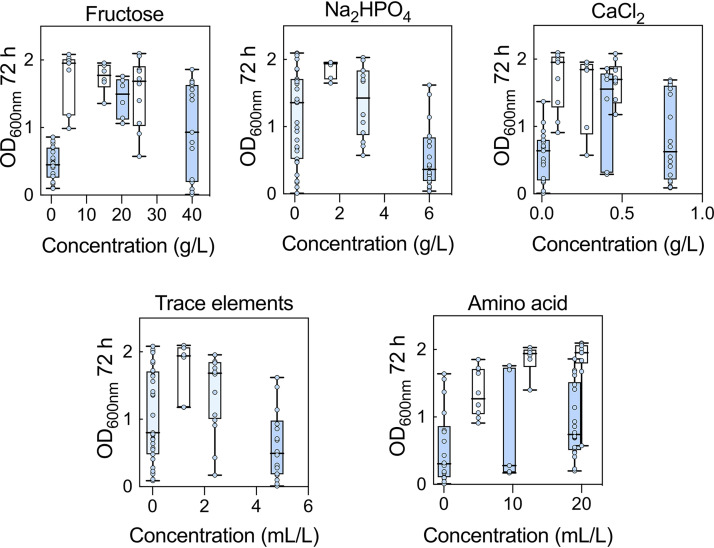
Combined data for DSD1 (blue boxes) and DSD2 (white boxes) for key medium components. Settings where the two DSDs were held at the same concentration are colored teal. Error bars indicate SEMs; *n *= 2 biological replicate arrays for DSD1 and DSD2.

A definitive screening design can force many of the sampling points collected to the edges of the design space. For this reason, we ran a second definitive screening design (DSD2) array (Table S3) in which the concentration ranges of the components were guided by the data from DSD1 and the factors under investigation were restricted to those that were highlighted as significant in DSD1 (Fig. 2). Examining the combined data for DSD1 and DSD2 did indeed confirm that the extreme concentrations of some of the components were detrimental to cell growth. For example, when the concentrations of fructose were at the highest and lowest values (40 and 0.5 g/liter, respectively) the OD_600_s at 72 h were both lower and more variable than when fructose was restricted to between 5 and 25 g/liter (Fig. 3). This indicates that maintaining fructose between 5 and 25 g/liter is key to establishing robust and reliable growth. Likewise, adjustments were made to the concentration ranges of the amino acids (5 and 20 ml/liter), CaCl_2_ (0.1 and 0.459 g/liter), and Na_2_HPO_4_ (0.1 and 3.05 g/liter). Other factors not identified as statistically significant were kept at the midpoint from DSD1, except NH_4_Cl, which was set at the lowest value because there was some evidence of a negative impact from the 2-level factor analysis (Fig. 2A).

**FIG 4:**
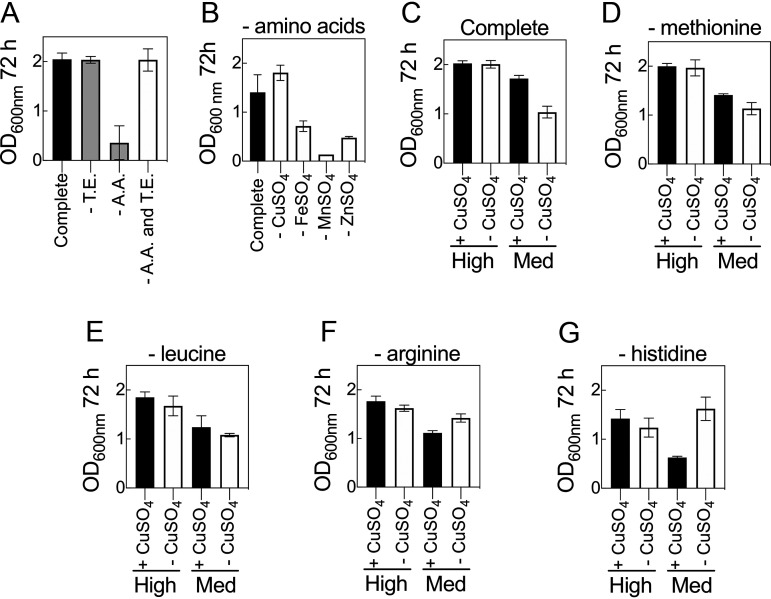
Interactions between trace elements and amino acids. (A) C. necator grown in a complete, chemically defined medium with either amino acids, trace elements, or both amino acids and trace elements excluded. (B) C. necator grown in a complete medium with each of the four trace elements excluded. (C to G) C. necator grown in a complete, chemically defined medium with or without CuSO_4_ and with each of the four amino acids excluded. Amino acid solution was added from 100× stock at 20 ml/liter (high) and 10 ml/liter (medium). Trace solution was added from a working concentration at 2.4 ml/liter. The concentrations of the ingredients in the amino acids and trace element solutions are as shown in Table 1. Error bars indicate SEMs; *n *= 2 biological replicates.

Examination of the distributions for DSD2 revealed that the medium compositions that generated the highest cell densities (OD_600_ > 2.0) were associated with low trace element concentrations and high amino acid content (Fig. S3). Moreover, given that both components are potential growth supplements, and each contained multiple ingredients, it was necessary to investigate the interaction between these two components to determine which ingredients were responsible for the observed interaction. To determine this, a medium that permits robust cell growth over 72 h was formulated and used as a control. It was observed that the absence of trace elements did not adversely affect growth of C. necator but that the absence of amino acids did (Fig. 4A). Interestingly, simultaneous exclusion of both amino acids and trace elements resulted in cell densities comparable to that of the control. We hypothesized that in the absence of one or more of the amino acids (methionine, histidine, leucine, and arginine), the presence of one or more of the components of the trace elements (CuSO_4_, FeSO_4_, MnSO_4_ and ZnSO_4_) inhibits growth of C. necator. This was tested first by formulating media without amino acids and withdrawing each trace element in turn (Fig. 4B). Under these conditions, media without CuSO_4_ but containing other trace elements resulted in growth comparable to that of the control, while all three formulations that contained CuSO_4_ had reduced cell densities. These observations support the hypothesis that CuSO_4_—in the absence of amino acids—inhibits C. necator growth. To determine which amino acid(s) interacts with CuSO_4_, a series of experiments were performed in which each amino acid was excluded in formulations with and without CuSO_4_. The first observation was that at high concentrations of amino acids (20 ml/liter), the presence or absence of CuSO_4_ did not affect growth (Fig. 4C). The second observation was that at medium concentrations of amino acids (10 ml/liter), growth was partially suppressed in the presence of CuSO_4_ but that this was exacerbated in the absence of CuSO_4_. Copper sulfate is therefore an important medium component and cannot simply be excluded from formulations. Similar growth responses were seen in experiments in which either methionine or leucine was excluded (Fig. 4D and E). If arginine or, most notably, histidine was removed from the medium, then the presence of CuSO_4_ impaired growth of C. necator (Fig. 4F and G). For media lacking histidine, this effect was also observable when all other amino acid levels were kept high (Fig. 4G). From these data we established two points: first, that histidine protects against the inhibitory effects of copper, and second, that CuSO_4_ is an important component of the media whose absence retards growth when amino acid content is restricted. Thus, the interaction between CuSO_4_ and histidine is a concentration-dependent positive interaction. Both CuSO_4_ and histidine concentrations must therefore be balanced for robust growth. Although formulations containing higher concentrations of amino acid (>10 ml/liter) can result in higher OD_600_s, such concentrations contributed ∼12% to growth in the absence of fructose as a carbon source, whereas under the same formulation, concentrations of ≤10 ml/liter of amino acid contributed <5% to growth. Hence, in subsequent experiments, amino acid concentration was set at 10 ml/liter and trace at ≤1 ml/liter.

### Final data augmentation.

**FIG 5:**
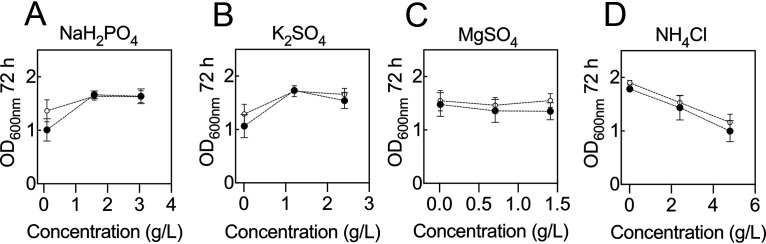
Reexamination of nonsignificant medium components. The impact of components not deemed statistically significant in DSD1 were reexamined under less extreme conditions. (A) NaH_2_PO_4_; (B) K_2_SO_4_; (C) MgSO_4_; (D) NH_4_Cl. Experiments were conducted at 5 g/liter (open circles) or 20 g/liter (closed circles) of fructose. Error bars indicate SEMs; *n *≥ 6.

With a greater understanding of which components and concentrations are required to formulate a medium supporting robust growth, we reinvestigated the roles that NaH_2_PO_4_, K_2_SO_4_, MgSO_4_, and NH_4_Cl have on the system. In the original DSD these factors were not identified as being statistically significant, but those experiments were conducted under conditions in which key components (e.g., fructose and amino acids) were at settings that have since been identified as resulting in poor or unreliable growth responses. We therefore reevaluated these factors under conditions in which Na_2_HPO_4_, CaCl_2_, trace element, and amino acid concentrations were not disruptive to cell growth (Table S4). Fructose was set at either 5 g/liter or 20 g/liter. The results indicated that these components did indeed affect growth rate when primary factors were not restricting growth (Fig. 5). Some of the suggestions from the original DSD were confirmed. Most notable was the observation that increasing NH_4_Cl had a detrimental effect on culture cell density. Sodium dihydrogen phosphate (NaH_2_PO_4_) and K_2_SO_4_ had some detrimental impact if concentrations were low, while MgSO_4_ concentrations were not significant. These results were true at both high and low concentrations of fructose.

### Modeling the medium formula-growth response landscape.

**FIG 6:**
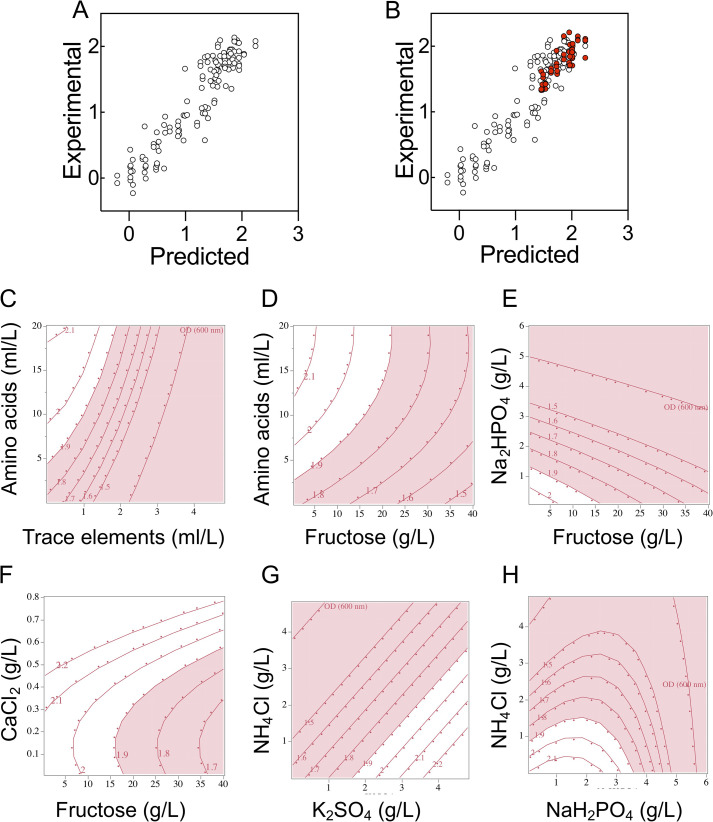
Experimental validation of model predictions in 48-well plate format and interactions of components of the defined media described by the least-squares model. (A) Model predicted values plotted against experimental data for the least-squares model (*r*^2^ = 0.87). (B) New experimental data for 1-ml culture volumes overlaid against the original least-squares model (*r*^2^ for new data only = 0.85). Open circles show the training data set; closed red circles show new data. Three biological replicates were assessed for each prediction. (C) Amino acid and trace element interactions. (D) Fructose and amino acid interactions. (E) Disodium phosphate and fructose interactions. (F) Calcium chloride and fructose interactions. (G) Ammonium chloride and potassium sulfate. (H) Ammonium chloride and monosodium phosphate. Each panel represents a two-way interaction. The red (pink) zone is an area where the OD_600_ at 72 h failed to reach 1.9, and the white zone is where it surpassed 1.9. Each contour grid line represents an OD_600_ increment of 0.1. All other medium components were kept at concentrations permitting high growth.

At this stage, 64 different variant formulations had been experimentally assessed in duplicate across three different experiments (25, 13, and 26 experimental runs). We then built a statistical model, trained against this data set, that could describe our understanding of how the cell cultures respond to changes in medium composition and predict performance in novel formulations. We performed two-level screening on all 128 runs. This identified a number of factors and factor interactions deemed significant for model projection. The screening did not highlight fructose or K_2_SO_4_ (as these had been at held concentrations that did not significantly impact growth during much of DSD2 and DSD3) but these terms were included manually in the model, as we knew they were important factors from the first DSD. These terms were used to construct a standard least-squares model. The least-squares model was able to describe the relationships within the data with good accuracy (Fig. 6A). Although the model was internally consistent, it was important to know if it could predict OD_600_ at 72 h in formulations it had not encountered during model training. We assessed 16 new formulations with sampling biased toward medium formulations that were predicted to be in the top 25% of medium performance. Each of these was assessed in triplicate and the resulting OD_600_ compared to predicted OD_600_ (Fig. 6B). As predicted by the model, all of the new formulations fell within the upper quartile of formula performance.

The model allowed us to visualize phenomena observed during the data collection phase of the investigation. For example, it visualizes the interaction between amino acids (specifically histidine) and trace elements (specifically copper) that is elucidated in Fig. 4. It shows that high concentrations of trace elements are detrimental to growth and that increasing the amino acid concentration can help mitigate the inhibitory effects of high concentrations of trace elements (Fig. 6C). The higher the concentration of trace elements included, the higher the amino acid concentration needs to be. Nevertheless, increasing the concentration of amino acids impacts other areas. A monotonic relationship was observed for fructose and amino acids; increasing the concentrations of both increased cell density (Fig. 6D). Although low concentrations of amino acids (≤5 ml/liter) may result in low cell density, this negative impact appears to be greater under high fructose concentrations. It follows that at 5 ml/liter of amino acids, reducing the fructose concentration to 5 g/liter will likely support higher cell density. Given that the amino acids present in the media are the only alternative source of carbon for growth, increasing the concentration of amino acids too much will impact interpretation of experiments designed to examine carbon utilization. A balance therefore needs to be established between fructose, amino acid, and trace element concentrations. The model also visualizes interactions between Na_2_HPO_4_ and fructose (Fig. 6E). Higher concentrations of Na_2_HPO_4_ results in lower cell densities—an effect that can be partially offset by decreasing fructose concentrations. The model also suggests that if CaCl_2_ is to be increased, fructose concentration must be increased and vice versa (Fig. 6F). Lastly, we had previously observed a negative effect of increasing NH_4_Cl concentrations (Fig. 5D). The model indicates that this can be mitigated by increasing the K_2_SO_4_ and/or NaH_2_PO_4_ concentration (Fig. 6G and H). Understanding the interactions between medium components is vital for predictions of culture performance and allows the experimenter to alter medium formulations for different experimental goals.

### Distinguishing between statistically significant and essential medium components.

**FIG 7:**
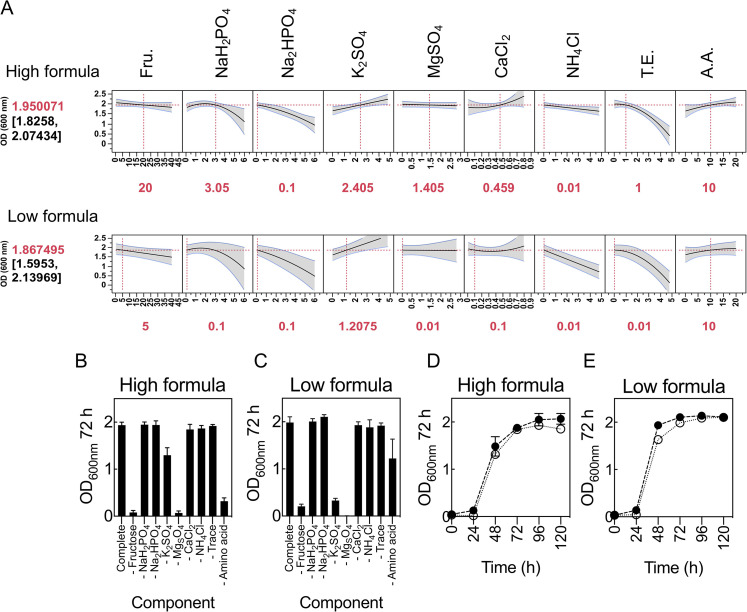
Determining the essential nature of each medium component. (A) Prediction profiler of growth at high- and low-concentration medium formulations. The model predicted rank and OD_600_ at 72 h (values in brackets) are shown on the left; medium settings for each component are shown in red underneath. All concentrations are in grams per liter except T.E. and A.A, which are in milliliters per liter. The output is 2 biological replicated arrays of definitive screening designs. (B and C) Impact of individually withdrawing each component on growth at high-concentration medium formulation (B) and low-concentration medium formulation (C). (D and E) Impact of simultaneously withdrawing some components (Na_2_HPO_4_, CaCl_2_, and NH_4_Cl) deemed nonessential on growth at high-concentration medium formulation (D) and low-concentration medium formulation (E). Error bars indicate SEMs; *n *= 3 biological replicates.

The growth model highlighted components and interactions that contribute significantly to growth. However, it is important to draw a distinction between what is statistically significant and what is essential. Determining the essential nature of components involves the conventional approach of withdrawing one factor individually during medium preparation. To ensure that the concentrations at which factors are held while individually withdrawing a component did not introduce bias on the essential nature of any component, two media were used as controls (Fig. 7A). One of these controls had every component maintained at low concentrations (low-concentration formula), while the other had every component maintained at high concentrations (high-concentration formula). Both controls were predicted to support robust growth of C. necator (Fig. 7A). When each component was individually withdrawn in both formulae, similar growth was observed, indicating that the concentrations at which components were held did not impact on the essential nature of any components of the media (Fig. 7B and C). Little growth was observed in media lacking fructose or MgSO_4_, whereas growth was significantly impaired in media lacking amino acids or K_2_SO_4_. The remaining components when individually withdrawn had little or no impact on growth. Therefore, it is established that fructose and MgSO_4_ are essential for cultivation of C. necator H16. Despite this, MgSO_4_ is not deemed statistically significant by the model (Fig. 2A). This is because under our experimental conditions, all concentrations of MgSO_4_ tested are likely in excess and do not limit growth. There is therefore the opportunity to reduce or increase MgSO_4_ concentrations if this were desirable. Lastly, amino acids and K_2_SO_4_ are considered important, while the remaining components are considered nonessential for growth. Interestingly, for both formulae, simultaneously withdrawing some of the nonessential components (Na_2_HPO_4_, CaCl_2_, and NH_4_Cl) resulted in similar growth patterns and gave OD_600_s comparable to that of the controls (Fig. 7D and E), arguing that cultivation of C. necator to high cell density is not nutritionally demanding—provided the main components of media are balanced.

Finally, CFU per milliliter were correlated against OD_600_ using control media predicted to support robust growth (Fig. 7A). This was performed to further confirm that OD_600_ is indeed appropriate for measuring C. necator cell density in defined media and that the OD_600_s observed throughout this study were ascribed to growth (viable cells) and not as a result of precipitation of components in media. Optical density and CFU per milliliter showed similar growth trends over 120 h of cultivation (Fig. S4).

### Model validation at greater volumes.

**FIG 8:**
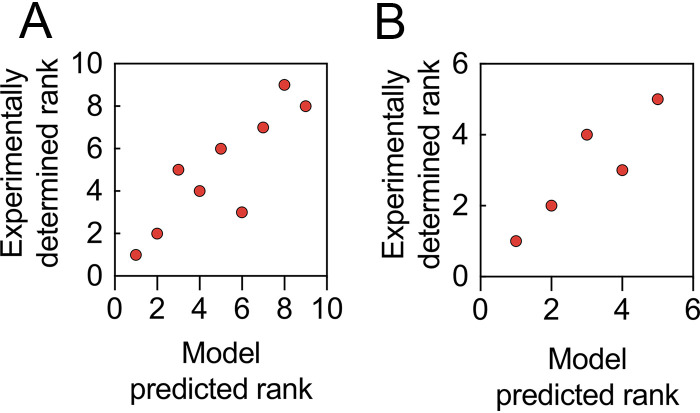
Experimental validation of model predictions at shake flask and bioreactor scales. (A) Spearman’s rank correlations (ρ = 0.87) between model-predicted rank and experimental data for 100-ml culture volumes in baffled and nonbaffled flasks. (B) Spearman’s rank correlations (ρ = 0.90) between model-predicted rank and experimental data for 1-liter bioreactor batch flask cultures.

To determine whether the growth model built using data collected from microtiter plates provides understanding of C. necator nutritional responses at larger culture volumes, we conducted two further model validation tests: first, at 100 ml in shake flasks, and second, at 1 liter using a bioreactor. Shake flask cultivations were carried out under identical conditions in baffled and nonbaffled flasks, using formulations randomly selected from an L32 fractional factorial design of experiment (Fig. S5). Growth (OD_600_) for each formula in both types of flasks was strikingly similar at every interval throughout the cultivation period. Most importantly, the growth ranks for baffled and nonbaffled flasks were identical. Spearman’s correlation showed a significant (*P < *0.05) relationship between predicted and actual growth ranks for both flask types; the correlation coefficient (ρ) is 0.87 (Fig. 8A). Next, two formulae from shake flask cultivation together with three additional formulae were cultivated in a 1-liter bioreactor (Fig. S6). Similarly, growth rank was predictable, with a significant relationship between predicted rank and actual growth rank (ρ = 0.90 [Fig. 8B]). During bioreactor cultivations, there were no significant changes in bioprocess parameters. Constant agitation at 200 rpm, with an airflow rate of 1 vvm (volume of air per volume of medium), was sufficient to maintain dissolved oxygen (dO_2_) above 20%. Although no base was added in all cultivations, the pH of the media did not drop below 4.5, the set point. Additionally, there was no correlation between pH of media (ρ = 0.23 and ρ = 0.52 for shake flask cultivation and bioreactor cultivation, respectively) prior to inoculation or after growth at 72 h (Fig. S7). Further, the predictive capability of the model was maintained irrespective of the aeration, whether the cultures were grown in baffled or nonbaffled flasks or in a bioreactor maintained at high oxygen concentrations.

## DISCUSSION

We developed a model trained against a structured data set for the cultivation of C. necator H16 in a chemically defined medium with fructose as the primary carbon source. Our approach identified significant growth factors and their effects on culture density at 72 h. Fructose, glucose, glycerol, and sucrose were used in the preliminary phase, with fructose considered the best carbon source supporting robust growth under heterotrophic conditions. While C. necator has been reported to have broad substrate range, its ability to utilize carbohydrates as a carbon source during heterotrophic growth appears to be limited to fructose and *N*-acetylglucosamine (8, 11, 29). Utilization of fructose by C. necator is most likely to occur via substrate import by an ATP-binding cassette (ABC)-type transporter, followed by catabolism via 2-keto-3-deoxy-6-phosphogluconate (KDPG), the Entner-Doudoroff pathway. The responsible genes, notably, a putative regulator (*frcR*), ribosome transporters (i.e., *frcA*, *frcC*, and *frcB* orthologs in Escherichia coli and Ralstonia solanacearum), and other essential genes facilitating such metabolism, are located on chromosome 2 inside gene clusters for glucose, 2-ketogluconate, and glucosamine catabolism (11). In contrast, phosphofructokinase and 6-phosphogluconate dehydrogenase, key enzymes of the Embden-Meyerhof-Parnas and oxidative pentose phosphate pathways, respectively, appear to be absent from the C. necator genome. It is therefore not surprising that glucose supported little or no growth, yet it has been reported that prolonged cultivation (>70 h) with glucose as the sole carbon source resulted in a mutant that was able to utilize glucose (30). The mutant acquired this ability by mutating the *N*-acetylglucosamine phosphotransferase system, which when deleted led to an inability to utilize glucose (30, 31). Glycerol supported low growth of C. necator. Such low growth was attributed to oxidative stress resulting from high levels of reactive oxygen species (ROS) formed as a result of elevated level of hydrogenases, leading to cell destruction by damaging DNA and other cell components (12).

Formulations of chemically defined media previously described for the cultivation of C. necator are typically prepared without amino acids (3, 7, 14, 16, 22, 32). In this study, bacterial growth was improved when a small number of amino acids (arginine, histidine, leucine, and methionine) were included in the medium. The data suggest that they serve as preferred sources of nitrogen compared to NH_4_Cl. Their inclusion resulted in a shorter lag phase than in media lacking amino acids. Although the effect of histidine is greater than those of methionine, arginine, and leucine, their action was synergistic. Interestingly, the model indicated that there was an interaction between fructose and amino acid content that indicates that the carbon-to-nitrogen balance must be maintained, irrespective of the actual values.

Metal ions, especially divalent cations of d-block transition metals, are important for bacterial growth, in which they act as metalloenzyme cofactors in living cells. Their presence in high concentrations, however, tends to be detrimental to cells (33). The Cu-histidine interaction highlighted by our model has been well studied across many forms of life (34–40). Copper is known to play diverse structural and catalytic functions owing to its ability to exist either in a reduced (Cu^+^) state with affinity for thiol and thioether groups or in an oxidized (Cu^2+^) state with more likely coordination for the oxygen or imidazole nitrogen group of amino acids, including histidine (41). The toxicity of Cu ions can range from catalysis of harmful redox reaction (when bound in weak sites) to disruption of enzyme functions (when bound in strong adventitious sites) (37). Bacteria respond to high levels of copper using different metalloregulators (39, 41). Under high concentrations, an integral membrane Cu^+^ transporter (P_1B_-type ATPase)—characterized by histidine-rich domains and conserved cytosine/histidine motifs within specific transmembrane domains—exports copper from the cytoplasm into the periplasm, where in Gram-negative bacteria further detoxification and exportation are carried out by other related enzymes, such as Cu^+^ oxidase, Cu chaperones, and nonspecific outer membrane metal cation transporters (39, 41, 42). Further, studies have reported that copper toxicity affected sugar (glucose) utilization in different microorganisms, and its growth inhibition on fungi (Neurospora crassa and Saccharomyces cerevisiae) was due to impairment/suppression of histidine biosynthesis (43, 44).

Does copper stress affect intracellular histidine availability or biosynthesis, or does the presence of sufficient histidine diminish the effect of copper stress? To address this, two important scenarios from the bacterium’s perspective need to be considered. First, is the bacterium responding to copper toxicity, or second, is the bacterium responding to low availability of histidine—which is more detrimental to growth? Our results suggest that the two scenarios are linked and are detrimental to growth. However, given that the bacterium can synthesize some of its amino acids sufficiently to sustain growth (11), and copper is established as an important trace metal for the bacterium’s growth (Fig. 4), it is more likely that something else increases the importance of histidine under copper stress, making histidine limitation detrimental to growth. Histidine is a strong metal-coordinating tridentate ligand with three potential metal-binding sites: the carboxylate oxygen group, the imidazole imido group, and the amino nitrogen group (35). The binding to these three sites depends on the Cu ions and the complexes formed. It appears that copper (Cu^+^ or Cu^2+^) stress does not impair histidine biosynthesis; rather, it increases the demand of histidine in the cell. This is supported by increased concentrations of histidine and other amino acids in the xylem sap of Brassica carinata under conditions of excess copper (38). It is unclear which Cu ion’s toxicity is diminished by histidine addition. Answering this question will require robust metallomic studies of C. necator, which will help shed light on the mechanism involved in copper export and trafficking in the microbe, a copper-loving bacterium. Further, it will help establish whether the toxicity is inside the cell (i.e., Cu^+^ toxicity) or outside the cell (i.e., Cu^2+^ toxicity). It is still unclear the exact role histidine plays, and with which form of Cu ions, and where in the cell this role is performed. What is clear is that histidine, and some other amino acids, acts like an enhancer or coordinator of Cu ion exportation to maintain copper homeostasis.

This study provides insight into the impact of medium formulations on growth and cell density of C. necator. The information provided, the small-scale automated experimentation and the statistical approaches undertaken in this study, will inform further efforts aimed at optimizing other C. necator responses. This includes the biosynthesis of polyhydroxyalkanoate, platform chemicals, proteins, and other products as well as future optimization of growth under lithoautotrophic conditions with CO_2_ and molecular hydrogen serving as carbon and energy sources, respectively.

## MATERIALS AND METHODS

### Bacterial strains.

Cupriavidus necator H16 (DSM 428) was obtained from the Deutsche Sammlung von Mikroorganismen und Zellkulturen GmbH (DSMZ; German Collection of Microorganisms and Cell Cultures). The bacterial strain was resuscitated on nutrient agar (peptone at 5 g/liter and meat extract at 3 g/liter) according to the supplier’s instructions and incubated at 30°C for 48 h.

### Chemicals.

Carbon sources (glucose, fructose, glycerol, and sucrose), salts (with exception of MgSO_4_·H_2_O and NH_4_Cl), trace metals, amino acids (histidine, leucine, and arginine) and some vitamins (thiamine, niacin, and pantothenic acid) were obtained from Sigma-Aldrich. The remainder of the vitamins, MgSO_4_·H_2_O, NH_4_Cl, and methionine were obtained from Duchefa Biochemie B.V., BDH Chemicals, and Formedium.

### Consideration of factors.

A comparison of the literature for the use of defined media for C. necator growth identified variety in both the nature and concentrations of macroelements and trace elements required for robust cell growth (Table S1). Therefore, components that served as factors for the DOE were carefully selected based on knowledge on general nutritional requirements for bacteria. The macroelements considered were C, N, O, P, Ca, Mg, and S, while trace elements were Cu, Zn, Fe, and Mn. These basic components correspond to the components of one of the existing CDM for C. necator (32). This medium (20 g/liter of fructose, 4 g/liter of NaH_2_PO_4_, 4.6 g/liter of Na_2_HPO_4_, 0.45 g/liter of K_2_SO_4_, 0.39 g/liter of MgSO_4_, 0.062 g/liter of CaCl_2_, and 0.5 g/liter of NH_4_Cl and 1 ml of trace solution containing 15 g/liter of FeSO_4_·7H_2_O, 2.4 g/liter of MnSO_4_·H_2_O, 2.4 g/liter of ZnSO_4_·7H_2_O, and 0.48 g/liter of CuSO_4_·5H_2_O) served as a starting point for the investigation. Additionally, a few amino acids and vitamins (45, 46) were added as factors to determine their effect on C. necator growth. Because adjusting the pH of media might result in the addition of extra ions, which, in turn, might impact interpretation of how components contribute to growth, the pH of each medium was not adjusted but was measured when appropriate. C. necator has a broad range of applications, including enzymes, platform chemicals, and biopolymer syntheses; however, optical density (OD_600_) was chosen as the output to gain detailed understanding on how the bacterium responds to different nutritional conditions. Such understanding would guide other process improvements involving C. necator as a production host.

### Preparation of media.

Stock solutions of glucose, fructose, sucrose, vitamins, amino acids, and each trace metal solution were filter sterilized through a 0.22-μm filter, while stock solutions of glycerol, NaH_2_PO_4_, Na_2_HPO_4_, MgSO_4_·H_2_O, NH_4_Cl, K_2_SO_4_, and CaCl_2_·2H_2_O were autoclaved. The trace element working solution contained 0.48 g/liter of CuSO_4_·5H_2_O (dissolved in 0.1 M HCl), 15 g/liter of FeSO_4_·7H_2_O (freshly prepared during each trace reconstitution from individual stock), and 2.4 g/liter each of MnSO_4_·H_2_O and ZnSO_4_·7H_2_O. A 100× amino acid stock solution contained 12.9 g/liter of arginine and 10 g/liter each of histidine, leucine, and methionine, while 1,000× vitamin stock solutions contained 0.1 g/liter of pyridoxine, 0.02 g/liter of folic acid, and 0.05 g/liter each of niacin, nicotinamide, pantothenic acid, riboflavin, and thiamine. Subsequently, medium components were added from individual stock solutions except for trace elements, which were added from reconstituted working solution. Forty-eight-well plates were used in all trials, and medium reconstitution in each well was carried out using an automated liquid handling system (Eppendorf epMotion M5073). All stock solutions were prepared using water as a solvent and were further diluted in sterile distilled water unless stated otherwise.

### Inoculum preparation and growth measurement.

For each experiment, 48-h colonies from LB agar were washed twice in sterile distilled water and diluted to a working inoculum concentration in the range of 10^8^ CFU/ml. The inoculum was further diluted 1:100 in wells containing medium. Forty-eight-well microtiter plates were incubated in a rotary incubator at 30°C and 180 rpm. Optical density (OD) at 600 nm was measured every 24 h using a Varioskan LUX multimode microplate reader (Thermo Scientific).

### Batch cultivation in a bioreactor system.

Large-scale cultivations were carried out in a batch mode using 1 liter of chemically defined media contained in 2-liter-capacity fermentors (Applikon ADI fermentation system). During the cultivations, pH was maintained above 4.5, temperature at 30°C, and agitation at 200 rpm. Dissolved oxygen (dO_2_) was maintained above 20% (1, 6) with airflow at 1 vvm (volume of air per volume of medium). No antifoam agents or base was added during cultivation. Starter culture media were of the same formulations as used in fermentors and were prepared by growing 100 ml of culture in 250-ml nonbaffled flasks to late exponential phase at 30°C and 200 rpm for 48 h. Following polarization and calibration of the dO_2_ probe, fermentors were inoculated with 10 ml (48 h) of starter culture and cultivations were monitored on-line and off-line over 72 h. Samples were taken every 24 h for off-line OD_600_ measurement.

### Data analyses.

Experimental designs were created using JMP Pro statistical software (version 13.0), and data from each experiment were analyzed using the same software. Graphics were generated using GraphPad Prism 7.0.

## Supplementary Material

Supplemental file 1
