# Supplementary material for: Applying Statistical Design of Experiments To Understanding the Effect of Growth Medium Components on Cupriavidus necator H16 Growth
Source: Appl Environ Microbiol. 2020 Aug 18;86(17):e00705-20. doi: 10.1128/AEM.00705-20 (PMC7440812; doi:10.1128/AEM.00705-20)
Supplement: Supplemental file 1 [file AEM.00705-20-s0001.pdf]

Supplementary Materials for

Applying statistical design of experiments (DOE) to understanding the effect of growth media components on *Cupriavidus necator* H16 growth

Christopher C. Azubuike[a,b], Martin G. Edwards[a], Angharad M. R. Gatehouse[a], Thomas P. Howard[a]#

[a]School of Natural and Environmental Sciences, Faculty of Science, Agriculture and Engineering, Newcastle University, Newcastle-upon-Tyne, NE1 7RX, United Kingdom

[b]Department of Microbiology, Faculty of Science, University of Port Harcourt, East-West Road, P.M.B. 5323, Choba, Port Harcourt, Rivers State, Nigeria

#corresponding author, Thomas P. Howard, email: Thomas.Howard@newcastle.ac.uk

Tel: +44(0)1208 48 54

Table S1 Previously described chemically defined media for the growth of *C. necator*.

Carbon	Phosphate buffer	Potassium	Magnesium	Calcium	Nitrogen	Trace elements	Reference
50 Fructose	1.5 $Na_2HPO_4.12H_2O$, 0.25 KH_2PO_4,		0.75 $MgSO_4.7H_2O$	0.015 $CaCl_2$	2.39 $(NH_4)_2SO_4$, 0.285 nitrilotriacetic acid, 28% 0.9 $Fe(NH_4)_2$(citrate)	0.00045 H_3BO_3, 0.0003 $CoCl_2$, 0.00015 $ZnSO_4.7H_2O$, 0.000045 $MnCl_2.4H_2O$, 0.000045 NaMoO4.2H_2O, 0.00003 $NiCl_2.6H_2O$ and 0.000015$CuSO_4.5H_2O$	(1)
0.5-1% Fructose	12 ml 1.1 M H3PO_4	0.45			0.1 or 0.01% NH_4Cl	24 mL/trace: 2.6 $CaCl_2.2H_2O$ 0.1 $MnSO_4.H_2O$, 0.1 ZnSO4.7H_2O, 0.02 $CuSO_4.5H_2O$, 0.015 $FeSO_4.7H_2O$	(2)
10-20 Fructose	1 KH_2PO_4; 11.1 $Na_2HPO_4.12H_2O$		0.2 $MgSO_4$		3 $(NH_4)_2SO_4$	9.7 $FeCl_3$, 7.8 $CaCl_2$, 0.156 $CuSO_4.5H_2O$), 0.119 $CoCl_2$, 0.118 $NiCl_2$ and 0.062 $CrCl_2$	(3)
2% v/v biodiesel-driven glycerol, 2 % w/v glycerol bottom and 1% v/v free fatty acids	6.7 $Na_2HPO_4.2H_2O$, 1.5 KH_2PO_4,		0.2 $MgSO_4.7H_2O$	0.01 $CaCl_2.2H_2O$	1 $(NH_4)_2SO_4$, 0.06 $Fe(NH_4)$2(citrate)	0.3 H_3BO_3, 0.2 $CoCl_2$, 0.1 $ZnSO_4.7H_2O$, 0.03 $MnCl_2.4H_2O$, 0.02 NaMoO4.2H_2O, 0.02 $NiCl_2.6H_2O$ and 0.01$CuSO_4.5H_2O$	(4)
20 Fructose	1.51 Na_2HPO_4, 2.65 KH_2PO_4,		0.3 $MgSO_4.7H_2O$			1.97 $FeCl_3$, 9.7 $CaCl_2.2H_2O$, 0.156 $CuSO_4.5H_2O$, 0.184 $CoCl_2.6H_2O$, 0.118 $NiCl_2.6H_2O$ and 0.062 $CrCl_3$	(5)
[a]2% Fructose, 2% sodium gluconate, 1% palm oil or 0.5% Tween-60	4 NaH_2PO_4, 4.6 Na_2HPO_4	0.45 K_2SO_4	0.39 $MgSO_4$	0.062 $CaCl_2$	0.5 NH_4Cl	15 $FeSO_4.7H_2O$, 2.4 $MnSO_4.H_2O$, 2.4 ZnSO4.7H_2O, 0.48 $CuSO_4.5H_2O$	(6, 7)
2-4 Fructose, 2 mL glycerol	1.5 KH_2PO_4, 9 Na_2HPO_4		0.2 $MgSO_4.7H_2O$	0.01 $CaCl_2.2H_2O$	2 NH_4Cl	0.05 $FeCl_3.6H_2O$, 0.019-0.190 $NiCl_2.6H_2O$	(8)

All concentrations are g/L unless otherwise indicated. Starting medium for DOE investigation ([a]).

Table S2 Definitive screening design array. A definitive screening design (DSD1) was developed to assess the impact of 10 ingredients found within the chemically defined media.

Media	Fru.	NaH_2PO_4	Na_2HPO_4	K_2SO_4	$MgSO_4$	$CaCl_2$	NH_4Cl	T.E.	A.A.	Vit.	OD1	OD2
1	20.25	0.1	0.1	0.01	0.01	0.01	0.01	0.01	0.05	0.01	1.06	1.37
2	20.25	6	6	4.8	2.8	0.8	4.8	4.8	19	4.8	1.14	1.62
3	0.5	3.05	0.1	4.8	0.01	0.01	0.01	4.8	19	4.8	0.52	0.47
4	40	3.05	6	0.01	2.8	0.8	4.8	0.01	0.05	0.01	0.09	0.78
5	0.5	6	3.05	0.01	2.8	0.01	0.01	0.01	19	4.8	0.77	0.71
6	40	0.1	3.05	4.8	0.01	0.8	4.8	4.8	0.05	0.01	-	-
7	40	6	0.1	2.41	2.8	0.01	4.8	4.8	19	0.01	0.93	0.69
8	0.5	0.1	6	2.41	0.01	0.8	0.01	0.01	0.05	4.8	0.11	0.28
9	0.5	6	0.1	4.8	1.41	0.01	4.8	0.01	0.05	0.01	0.80	0.64
10	40	0.1	6	0.01	1.41	0.8	0.01	4.8	19	4.8	1.62	1.48
11	0.5	6	6	0.01	2.8	0.41	0.01	4.8	0.05	0.01	0.29	0.32
12	40	0.1	0.1	4.8	0.01	0.41	4.8	0.01	19	4.8	1.86	1.41
13	0.5	6	6	4.8	0.01	0.8	2.41	0.01	19	0.01	0.41	0.55
14	40	0.1	0.1	0.01	2.8	0.01	2.41	4.8	0.05	4.8	-	0.01
15	0.5	0.1	6	4.8	2.8	0.01	4.8	2.41	0.05	4.8	0.17	0.43
16	40	6	0.1	0.01	0.01	0.8	0.01	2.41	19	0.01	1.69	1.67
17	0.5	0.1	0.1	4.8	2.8	0.8	0.01	4.8	9.53	0.01	0.17	0.28
18	40	6	6	0.01	0.01	0.01	4.8	0.01	9.53	4.8	-	0.19
19	0.5	0.1	0.1	0.01	2.8	0.8	4.8	0.01	19	2.41	0.48	0.70
20	40	6	6	4.8	0.01	0.01	0.01	4.8	0.05	2.41	-	0.04
21	20.25	3.05	3.05	2.41	1.41	0.41	2.41	2.41	9.53	2.41	1.76	1.70
22	0.5	0.1	6	0.01	0.01	0.01	4.8	4.8	19	0.01	0.86	0.71
23	0.5	6	0.1	0.01	0.01	0.8	4.8	4.8	0.05	4.8	0.10	0.19
24	40	6	0.1	4.8	2.8	0.8	0.01	0.01	0.05	4.8	1.64	1.56
25	40	0.1	6	4.8	2.8	0.01	0.01	0.01	19	0.01	0.20	0.23

A definitive screening design was developed to assess the impact of 10 ingredients found within the chemically defined media. All concentrations are in g/L except trace elements, amino acids and vitamins which are mL/L. Trace element working concentration contained (g/L): 15 $FeSO_4.7H_2O$, 2.4 $MnSO_4.H_2O$, 2.4 $ZnSO_4.7H_2O$, and 0.48 $CuSO_4.5H_2O$. A 100x stock amino acid mix contained (g/L): 12.9 arginine, and 10 each of histidine, leucine and methionine. A 1000x vitamin stock contained (g/L): 0.1 pyridoxine, 0.02 folic acid, 0.05 each of thiamine, riboflavin, niacin, pantothenic acid and nicotinamide. Abbreviations: Fru., fructose, T.E. trace element mixture; A.A., amino acid mixture; Vit., vitamin mixture. The DSD was performed in n = 2 biological replicates.

Table S3 Definitive Screening Design 2. A second definitive screening design (DSD2) was performed to assess the impact of the five ingredients identified as contributing significantly to *C. necator* growth.

Media	Fru.	NaH_2PO_4	Na_2HPO_4	K_2SO_4	$MgSO_4$	$CaCl_2$	NH_4Cl	T.E.	A.A.	OD1	OD2
1	15	3.05	0.1	2.41	1.41	0.01	0.01	0.01	5	1.71	1.36
2	15	3.05	3.05	2.41	1.41	0.46	0.01	2.41	20	1.84	1.67
3	25	3.05	1.58	2.41	1.41	0.46	0.01	0.01	5	1.72	1.65
4	5	3.05	1.58	2.41	1.41	0.01	0.01	2.41	20	1.95	1.95
5	5	3.05	0.1	2.41	1.41	0.28	0.01	2.41	5	1.85	0.99
6	25	3.05	3.05	2.41	1.41	0.28	0.01	0.01	20	0.57	1.84
7	5	3.05	3.05	2.41	1.41	0.46	0.01	1.28	5	1.18	1.18
8	25	3.05	0.1	2.41	1.41	0.01	0.01	1.28	20	2.10	2.06
9	5	3.05	3.05	2.41	1.41	0.01	0.01	0.01	12.50	2.03	1.99
10	25	3.05	0.1	2.41	1.41	0.46	0.01	2.41	12.50	1.86	1.40
11	15	3.05	1.58	2.41	1.41	0.28	0.01	1.28	12.50	1.92	1.96
12	5	3.05	0.1	2.41	1.41	0.46	0.01	0.01	20	2.01	2.08
13	25	3.05	3.05	2.41	1.41	0.01	0.01	2.41	5	1.06	0.91

All concentrations are in g/L except trace elements and amino acids, which are mL/L. Trace element working concentration contained (g/L): 15 $FeSO_4.7H_2O$, 2.4 $MnSO_4.H_2O$, 2.4 $ZnSO_4.7H_2O$, and 0.48 $CuSO_4.5H_2O$. A 100x stock amino acid mix contained (g/L): 12.9 arginine, and 10 each of histidine, leucine and methionine. Abbreviations: Fru., fructose, T.E., trace element mixture; A.A., amino acid mixture. The DSD2 array was performed in duplicate.

Table S4 Definitive Screening Design 3. A final set of experiments were performed to re-assess the impact of components not deemed significant in the first DSD.

Media	Fru.	NaH_2PO_4	Na_2HPO_4	K_2SO_4	$MgSO_4$	$CaCl_2$	NH_4Cl	T.E.	A.A.	OD1	OD2
1	5	0.1	0.1	0.01	0.01	0.46	4.8	1	10	0.13	0.17
2	5	0.1	0.1	1.21	1.41	0.46	0.01	1	10	1.79	1.74
3	5	0.1	0.1	2.41	0.01	0.46	0.01	1	10	1.64	1.71
4	5	0.1	0.1	0.01	1.41	0.46	2.41	1	10	0.95	0.57
5	5	3.05	0.1	1.21	0.01	0.46	4.8	1	10	1.85	1.76
6	5	3.05	0.1	0.01	0.71	0.46	0.01	1	10	1.86	1.65
7	5	3.05	0.1	2.41	1.41	0.46	0.01	1	10	1.89	1.88
8	5	1.56	0.1	0.01	0.01	0.46	0.01	1	10	1.76	1.92
9	5	3.05	0.1	0.01	1.41	0.46	4.8	1	10	0.84	0.79
10	5	0.1	0.1	2.41	0.71	0.46	4.8	1	10	0.66	0.73
11	5	1.56	0.1	2.41	1.41	0.46	4.8	1	10	1.51	1.55
12	5	3.05	0.1	2.41	0.01	0.46	2.41	1	10	1.95	1.89
13	5	1.56	0.1	1.21	0.71	0.46	2.41	1	10	1.49	1.76
14	20	0.1	0.1	0.01	0.01	0.46	4.8	1	10	0.15	0.81
15	20	0.1	0.1	1.21	1.41	0.46	0.01	1	10	1.86	2.09
16	20	0.1	0.1	2.41	0.01	0.46	0.01	1	10	2.09	1.92
17	20	0.1	0.1	0.01	1.41	0.46	2.41	1	10	1.66	0.95
18	20	3.05	0.1	1.21	0.01	0.46	4.8	1	10	1.79	1.52
19	20	3.05	0.1	0.01	0.71	0.46	0.01	1	10	1.71	1.90
20	20	3.05	0.1	2.41	1.41	0.46	0.01	1	10	2.05	1.73
21	20	1.56	0.1	0.01	0.01	0.46	0.01	1	10	1.81	1.85
22	20	3.05	0.1	0.01	1.41	0.46	4.8	1	10	1.08	0.98
23	20	0.1	0.1	2.41	0.71	0.46	4.8	1	10	1.17	0.96
24	20	1.56	0.1	2.41	1.41	0.46	4.8	1	10	1.65	1.48
25	20	3.05	0.1	2.41	0.01	0.46	2.41	1	10	1.74	1.80
26	20	1.56	0.1	1.21	0.71	0.46	2.41	1	10	1.65	1.39

All concentrations are in g/L except trace elements and amino acids, which are mL/L. Trace element working concentration contained (g/L): 15 FeSO4.7H2O, 2.4 MnSO4.H2O, 2.4 ZnSO4.7H2O, and 0.48 CuSO4.5H2O. A 100x stock amino acid mix contained (g/L): 12.9 arginine, and 10 each of histidine, leucine and methionine. Abbreviations: Fru., fructose, T.E., trace element mixture; A.A., amino acid mixture. The DSD array was performed in duplicate.

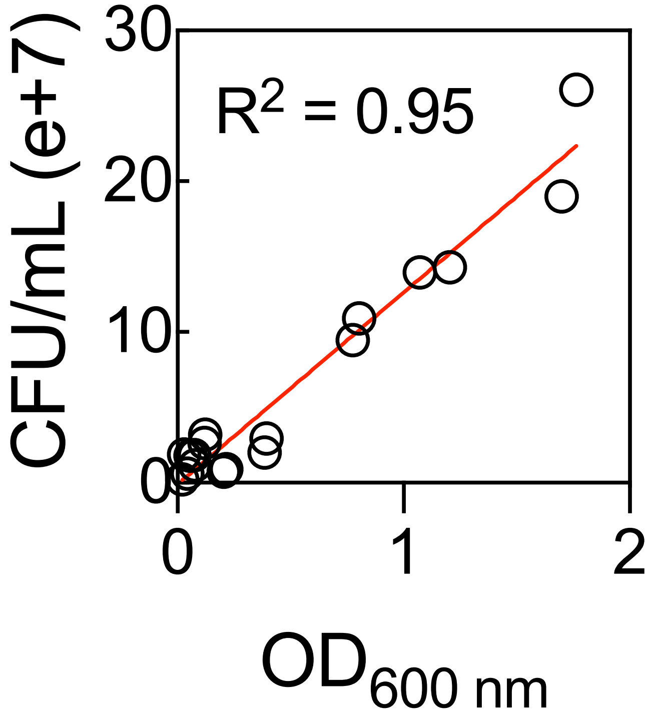

FIG S1 Correlation of colony forming unit per millilitre (CFU/mL) against optical density (OD_{600nm}). Data are for n = 3 biological replicates.

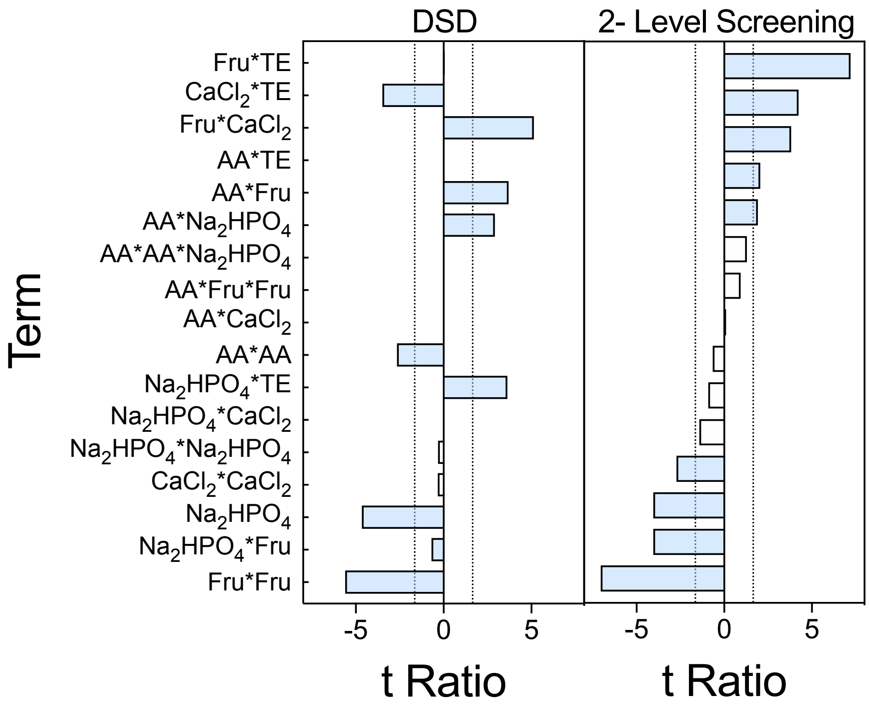

FIG S2 Two-way interaction between components. The comparative lengths of the t-ratios for factor interaction are shown. Bars extending to the right have a positive impact on growth at high concentration, those extending to the left have a negative impact on growth at high concentration. The broken vertical lines indicate threshold ($t < -1.65$ or > 1.65) level at 90% confidence level; terms (interaction) surpassing this threshold is deemed to have significant effect on the output, OD_{600nm}. Terms deemed significant for model projection are highlighted (blue). Analysis is based on two biological replicated arrays (Table S2).

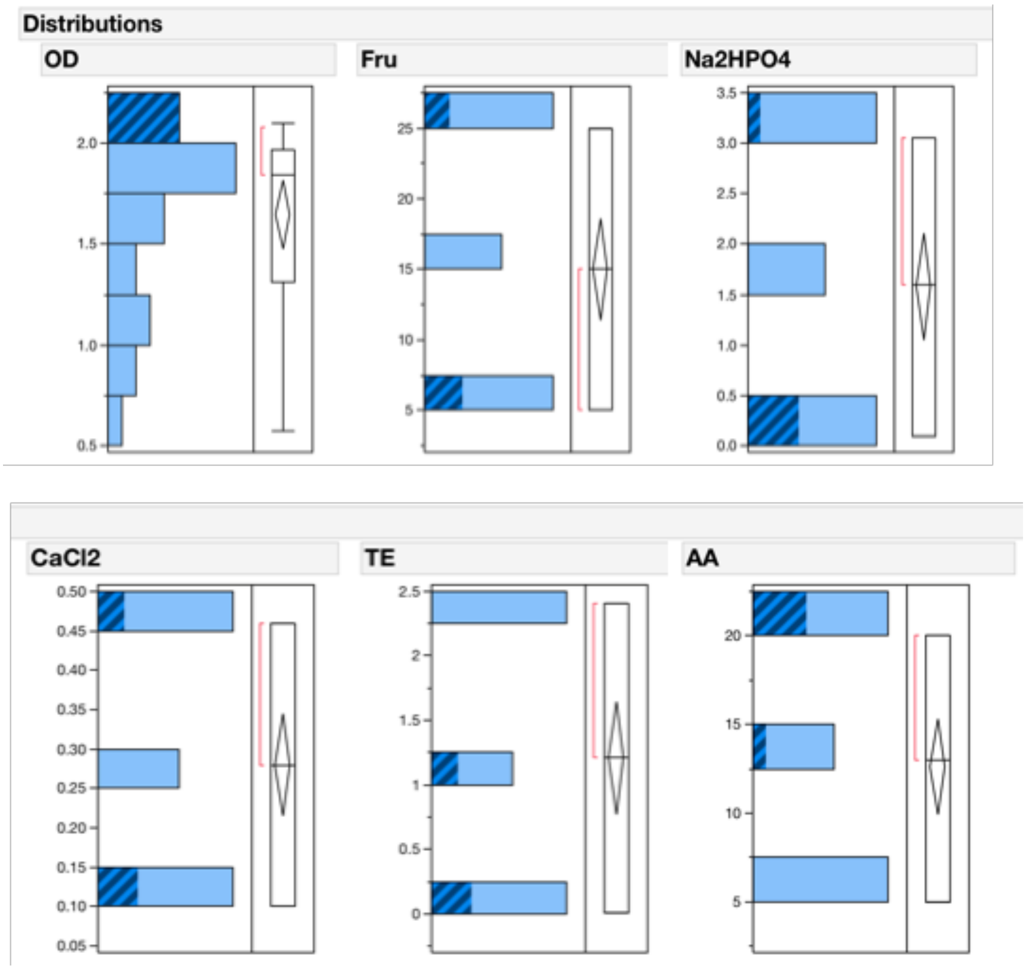

FIG S3 Distributions of data for Definitive Screening Design 2. Highlighted in blue crosshatch are the settings that resulted in the greatest OD_{600nm} at 72 h for DSD2. Fructose, Na_2HPO_4 and $CaCl_2$ may be at either the highest or lowest concentrations (g/L), whereas trace elements and amino acid concentrations are found at the lowest or highest concentrations (mL/L) respectively.

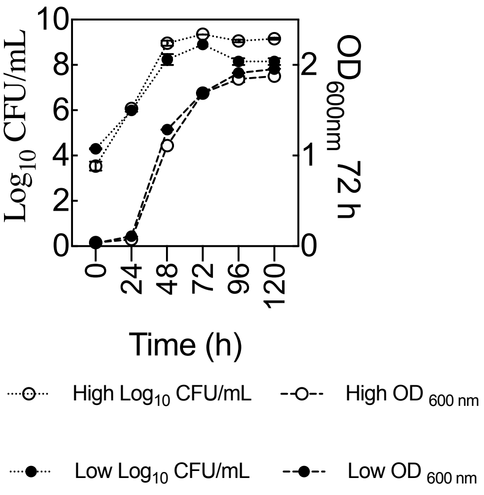

FIG S4 Validation of OD_{600nm} as surrogate for growth measurement. Viable cells (cfu/mL) of *C. necator* H16 correlated with optical density (OD_{600nm}) over prolonged cultivation. Experiment was performed at low and high concentration media formulations, guided by the growth model prediction (Fig. 7A). Cell were grown on defined media and plated on LB agar and incubated as appropriate for *C. necator*. Error bars are S.E. Mean, n = 3 biological replicates.

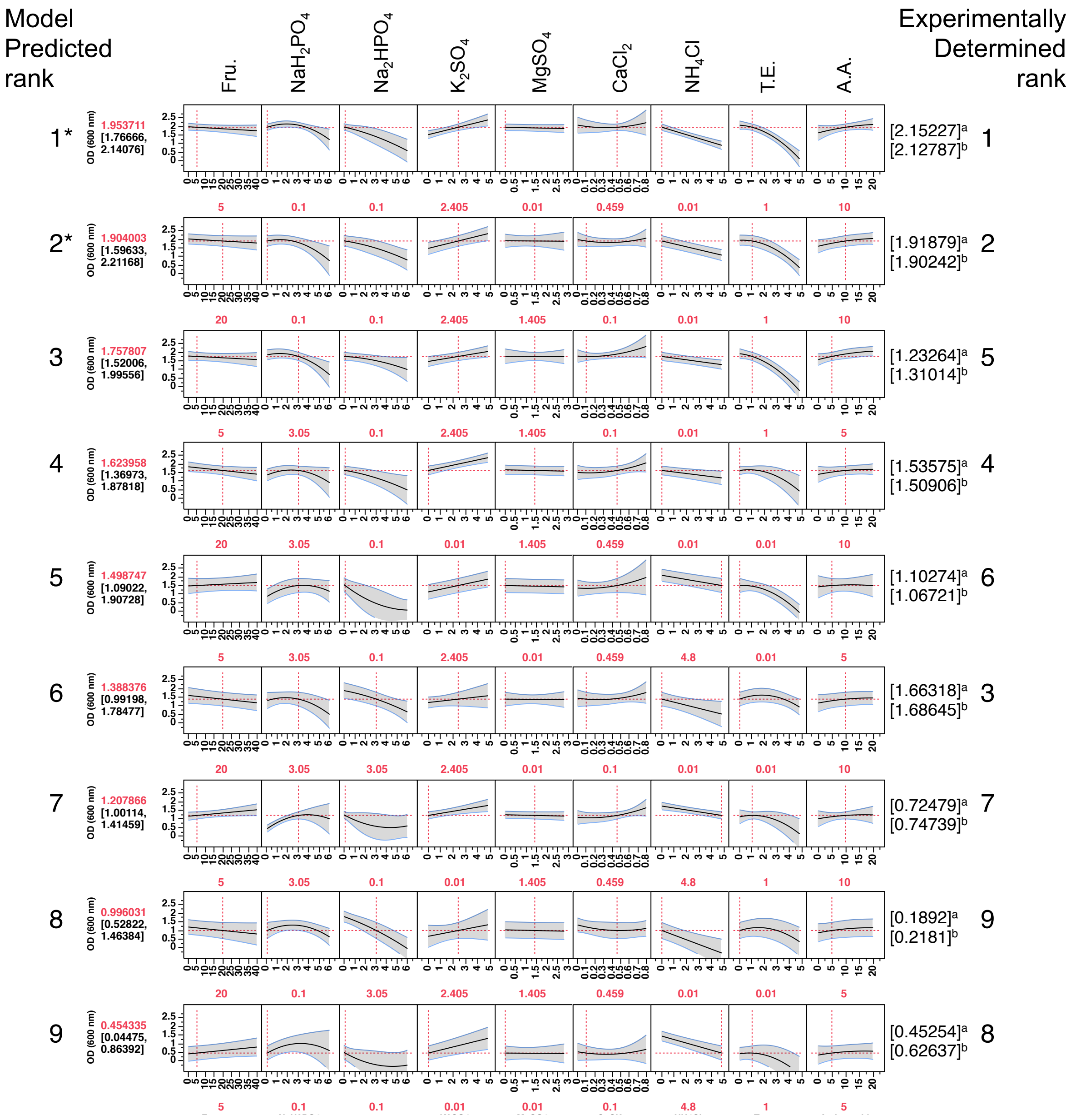

FIG S5 One hundred millilitre validation in shake flasks. Nine different formulations were assessed at 100 mL culture volumes. For each row, the model predicted rank and OD_{600nm} 72 h (values in brackets) are indicated on the left, media settings for each component are indicated in red underneath, the measured OD_{600nm} 72 h for two cultivation experiments (values in brackets) and experimentally determined rank are indicated on the right. Non-baffled flask cultivations (a); baffled flask cultivations (b); included in bioreactor cultivations and subsequently served as controls (*). All concentrations are in g/L except T.E. and A.A, which are in mL/L. Abbreviations: Fru., fructose; T.E., trace element mixture; A.A., amino acid mixture. The output is $n = 2$ biological replicated arrays of definitive screening designs (DSDs).

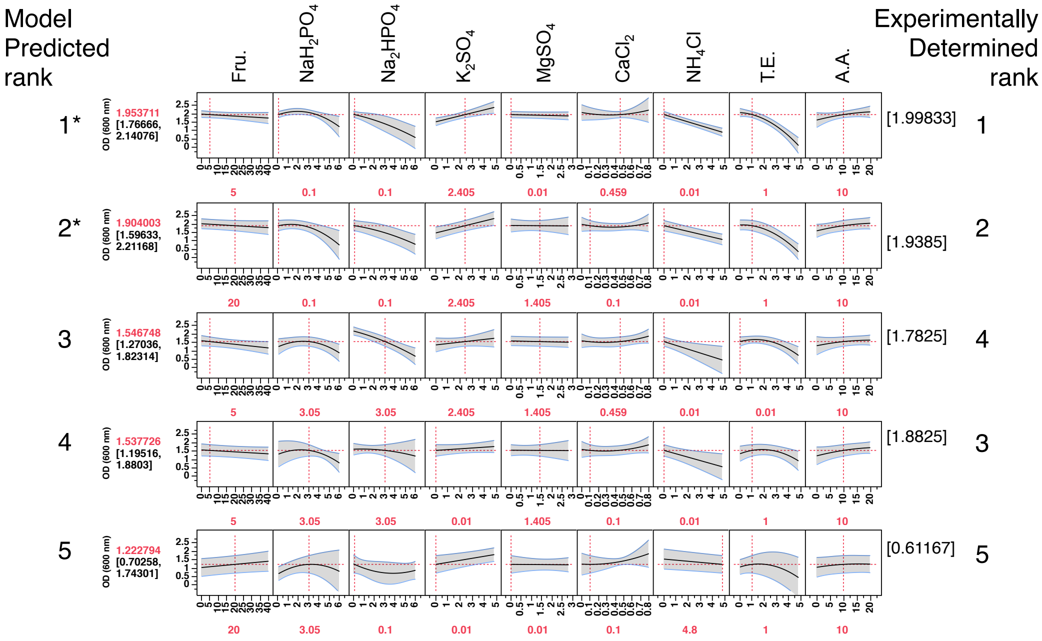

FIG S6 One litre (1 L) validation. Five different formulations were assessed at 1 L culture volumes. For each row, the model predicted rank and OD_{600nm} 72 h (values in brackets) are indicated on the left, media settings for each component are indicated in red underneath; the measured OD_{600nm} 72 h (values in brackets) and experimentally determined rank are indicated on the right. Controls (*). All concentrations are in g/L except T.E. and A.A, which are in mL/L. Abbreviations: Fru., fructose; T.E., trace element mixture; A.A., amino acid mixture.

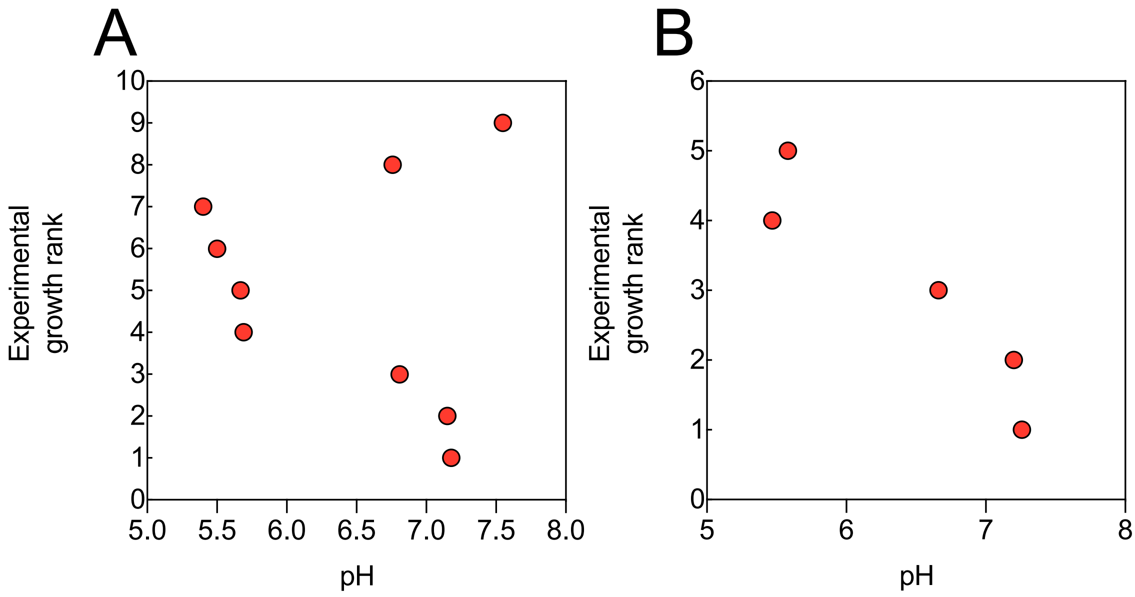

FIG S7 Effect of pH on growth. Spearman's correlation of experimentally determined growth ($OD_{600\ nm}$) and pH after 72 h. **A.** Shake flask cultivation (ρ = 0.23). **B.** Bioreactor cultivation (ρ = 0.52).

References

1. Grousseau E, Lu J, Gorret N, Guillouet SE, Sinskey AJ. 2014. Isopropanol production with engineered *Cupriavidus necator* as bioproduction platform. Appl Microbiol Biotechnol 98:4277–4290.

2. Müller J, MacEachran D, Burd H, Sathitsuksanoh N, Bi C, Yeh YC, Lee TS, Hillson NJ, Chhabra SR, Singer SW, Beller HR. 2013. Engineering of *Ralstonia eutropha* H16 for autotrophic and heterotrophic production of methyl ketones. Appl Environ Microbiol 79:4433–4439.

3. Obruca S, Marova I, Stankova M, Mravcova L, Svoboda Z. 2010. Effect of ethanol and hydrogen peroxide on poly(3-hydroxybutyrate) biosynthetic pathway in *Cupriavidus necator* H16. World J Microbiol Biotechnol 26:1261–1267.

4. Sharma PK, Fu J, Spicer V, Krokhin O V., Cicek N, Sparling R, Levin DB. 2016. Global changes in the proteome of *Cupriavidus necator* H16 during poly-(3-hydroxybutyrate)

synthesis from various biodiesel by-product substrates. AMB Express 6:36.

5. Aneja KK, Ashby RD, Solaiman DKY. 2009. Altered composition of *Ralstonia eutropha* poly(hydroxyalkanoate) through expression of PHA synthase from Allochromatium vinosum ATCC 35206. Biotechnol Lett 31:1601–1612.

6. Marc J, Grousseau E, Lombard E, Sinskey AJ, Gorret N. 2017. Over expression of GroESL in *Cupriavidus necator* for heterotrophic and autotrophic isopropanol production. Metab Eng 42:74–84.

7. Li H, Opgenorth PH, Wernick DG, Rogers S, Wu T, Higashide W, Malati P, Huo Y, Cho KM, Liao JC. 2012. Integrated electromicrobial conversion of CO2 to higher alcohols. Science (80-) 335:1596.

8. Cramm R. 2008. Genomic view of energy metabolism in *Ralstonia eutropha* H16. J Mol Microbiol Biotechnol 16:38–52.
